# Prevalence of Emotional Eating and Its Relationship with Anthropometric and Biochemical Indicators in University Students

**DOI:** 10.3390/nu18050853

**Published:** 2026-03-06

**Authors:** Adriana Aguilar-Galarza, Miriam Hernández-Meza, Karla Carmina Rojas-Saavedra, Karina de la Torre-Carbot, Cristina Elizabeth Fuente-González, Jorge Luis Chávez-Servín

**Affiliations:** 1 Programa de Maestría en Nutrición Clínica Integral, Facultad de Ciencias Naturales, Universidad Autónoma de Queretaro, Av. De las Ciencias S/N, Juriquilla, Querétaro 76230, Mexico; beatriz.aguilar@uaq.edu.mx (A.A.-G.); karina.delatorre@uaq.edu.mx (K.d.l.T.-C.); cristina.fuente@uaq.mx (C.E.F.-G.); 2 Servicio Universitario de Salud, Secretaría de Vinculación y Servicios Universitarios, Universidad Autónoma de Querétaro, Carr. A Chichimequillas S/N, Ejido Bolaños, Querétaro 76140, Mexico; 3 Tecnologico de Monterrey, Escuela de Medicina y Ciencias de la Salud, Epigmenio González 500, Fracc, San Pablo, Querétaro 76130, Mexico; karlarojas@tec.mx

**Keywords:** emotional eating, university students, anthropometry, metabolic risk, Garaulet EEQ, behavioral nutrition, young adults, obesity risk factors

## Abstract

**Background/Objectives**: Emotional eating is a behavioral pattern in which individuals increase food intake in response to emotional states rather than physiological hunger. University students are particularly vulnerable due to academic stress, lifestyle changes, and a food environment dominated by highly palatable options. Although emotional eating has been associated with adiposity and metabolic alterations, evidence in Mexican university populations remains limited. This study aimed to estimate the prevalence of emotional eating and to examine its association with anthropometric and biochemical indicators in students from the Universidad Autónoma de Querétaro (UAQ). **Methods**: A cross-sectional study was conducted among 670 first-year university students participating in the SU SALUD-UAQ clinical evaluation. Emotional eating was assessed using the 10-item Emotional Eater Questionnaire (EEQ). Anthropometric measures (body mass index BMI, body fat percentage, and waist circumference) and biochemical markers (triglycerides, total cholesterol, HDL cholesterol, and glucose) were obtained through standardized clinical procedures. Associations were evaluated using multivariable linear and logistic regression models adjusted for sex, age, physical activity level, sleep duration, stress, and socioeconomic status. **Results**: The prevalence of emotional eating categories was as follows: non-emotional (33.5%), low emotional (31.1%), emotional (27.6%), and highly emotional (7.8%). Higher EEQ scores were independently associated with greater BMI, body fat percentage, and waist circumference in both sexes. In women, emotional eating was also independently associated with less favorable lipid profiles. In addition, students classified as emotional or highly emotional eaters showed higher odds of general and abdominal obesity, particularly among women. **Conclusions**: Emotional eating is highly prevalent among Mexican university students and is independently associated with increased adiposity in both sexes and with altered lipid profiles in women. These findings highlight the relevance of integrating emotional regulation strategies into university health programs as a component of comprehensive health promotion approaches aimed at addressing emotional eating and its associated anthropometric and metabolic correlates in young adults.

## 1. Introduction

Eating behavior can be influenced by internal factors, such as emotions, stress, impulsivity, and affective disorders, as well as by external factors, including the university food environment, food advertising, irregular schedules, and sedentary lifestyles. Sustained exposure to ultra-processed foods, sleep deprivation, and elevated academic stress has been shown to promote the use of food as a coping strategy, thereby facilitating dysregulated eating patterns such as binge eating and emotional eating [1]. Emotional eating is defined as the tendency to modify—most commonly to increase—food intake in response to emotional states rather than physiological signals of hunger or satiety [2]. Traditionally, emotional eating has been described as occurring in response to negative emotions such as stress, anxiety, sadness, or loneliness. However, recent evidence also distinguishes positive emotional eating, whereby pleasant emotional states (e.g., joy, celebration, or euphoria) may trigger episodes of overeating, particularly involving energy-dense and highly palatable foods [3].

From a health psychology perspective, emotional eating is understood as a maladaptive coping strategy associated with difficulties in emotional regulation, attentional biases toward food-related stimuli, and impaired interoceptive awareness, which may hinder the ability to distinguish physiological hunger from emotional states [4,5]. Eating behavior is a multifactorial construct shaped by the interaction of biological, psychological, and sociocultural processes that influence food choice, intake, and consumption patterns.

Accumulating evidence suggests that emotional regulation, perceived stress, and body-related perceptions play a central role in shaping eating behavior, particularly among university students, a population exposed to significant life transitions and high academic demands [1].

At the international level, the prevalence of emotional eating is high. Among adults, it has been estimated that approximately 38% report episodes of emotional eating at least once per month, and nearly half report engaging in emotional eating on a weekly basis [2,5]. In university populations across different countries, recent studies indicate that approximately 30% to 40% of students are classified as emotional or highly emotional eaters, with higher emotional eating scores consistently associated with increased BMI, anxiety–depressive symptomatology, and academic stress [6,7,8]. Moreover, a recent meta-analysis in university students has documented that emotional eating is associated with higher perceived stress and poorer diet quality, highlighting this behavior as a relevant mechanism during the transition to adulthood [9,10].

In Mexico, eating behavior is strongly shaped by sociocultural context, including the meanings attributed to foods and their use within social settings for both conviviality and emotional regulation [4]. Studies using instruments developed in Mexican populations have shown that higher body weight is associated with a greater use of food to modulate the intensity or duration of emotional experiences [11]. Among Mexican university students, empirical models of emotional eating have identified that variables such as negative affect, weight concern, sociocultural pressure, and characteristics of the university environment are integrated into a complex construct associated with risky eating behaviors [12]. In addition, studies conducted in Mexico City have shown that emotional eating mediates the relationship between depressive symptoms and the frequent consumption of unhealthy foods among university students [13,14]. University students constitute a particularly vulnerable population, as entry into university is accompanied by substantial lifestyle changes, including increased academic workload, alterations in sleep schedules, reduced physical activity, increased availability of ultra-processed foods, and, frequently, residential and economic independence [7,15]. This stage of life also coincides with a high prevalence of overweight and obesity, underscoring the relevance of examining emotional eating patterns in relation to anthropometric and metabolic health indicators in this population [16,17].

Emotional eating has been assessed using various instruments, including the Three-Factor Eating Questionnaire (TFEQ), the Dutch Eating Behavior Questionnaire (DEBQ), the Emotional Eating Scale (EES), the Eating and Appraisal Due to Emotions and Stress Questionnaire (EADES), as well as tools developed specifically for Mexican populations [4,12,18,19]. However, due to its simplicity, rapid administration, and robust psychometric properties, the Garaulet EEQ has become a widely used instrument in both research and clinical settings. The EEQ consists of 10 items and classifies individuals into four categories (non-emotional, low emotional, emotional, and highly emotional eaters) based on the relationship between emotional states and food intake [20]. The original validation study demonstrated high internal consistency and construct validity, while subsequent validations in adults with overweight and obesity in Mexico and Colombia have confirmed its factorial structure, reliability, and applicability in Latin American populations [21,22].

Studies in individuals with obesity have examined the relationship between dysfunctional eating behaviors—including emotional eating—and cardiometabolic parameters, reporting associations with insulin resistance indices, lipid alterations, and cardiovascular risk markers [23,24,25]. However, in university populations, most studies have focused primarily on psychological outcomes (e.g., stress, anxiety, and depressive symptoms) or dietary patterns, with limited integration of objective health indicators.

Importantly, studies that simultaneously assess emotional eating using a validated instrument together with anthropometric indicators (such as BMI, body fat percentage, and waist circumference) and fasting biochemical markers of cardiometabolic risk (including triglycerides, cholesterol fractions, and glucose) remain scarce in Latin American university populations. This gap is particularly relevant, as early adulthood represents a critical period for the consolidation of eating behaviors and the early emergence of metabolic alterations that may track into later life.

In addition, few studies in this context have examined these associations using multivariable analytical approaches that account for relevant behavioral and sociodemographic factors during early adulthood. In this context, the present study contributes novel evidence by evaluating emotional eating using a validated instrument and linking it to standardized anthropometric and biochemical measurements within a large cohort of Mexican university students participating in an institutional health program. Importantly, the use of multivariable linear and logistic regression models allows the assessment of independent associations between emotional eating and adiposity- and metabolism-related indicators, beyond simple descriptive or unadjusted analyses.

Therefore, the aim of this study was to estimate the prevalence of emotional eating among students at the UAQ and to examine its independent associations with anthropometric and biochemical indicators after adjustment for key behavioral and sociodemographic factors. By focusing on a young adult population in a Latin American context, this work seeks to extend existing literature and provide context-specific evidence relevant for future preventive strategies in university health settings.

## 2. Materials and Methods

### 2.1. Study Design

This study employed a descriptive cross-sectional design. Data were collected during the comprehensive health assessment of first-year students at the UAQ, conducted through the University Health Service (SU SALUD-UAQ). Given the cross-sectional nature of the study, all measurements were obtained at a single time point without any intervention, allowing for the characterization of health status and emotional eating behaviors in the evaluated population. The study was carried out during the 2025 academic year.

### 2.2. Population and Sample

The study population consisted of first-year students from the UAQ who participated in the Comprehensive Clinical Examination conducted by the University Health Service (SU SALUD-UAQ). The sample included students who voluntarily attended the clinical evaluation during the admission period. Participants were recruited from multiple university campuses, including both metropolitan and regional sites, allowing for broad representation of the institution’s territorial diversity. Students from several academic units participated, including the Faculties of Natural Sciences, Political and Social Sciences, Philosophy, Computer Science, Engineering, Languages and Literature, and Chemistry, ensuring representation of diverse areas of knowledge. Inclusion criteria comprised male and female students aged 18 to 28 years, enrolled at any campus or faculty, who agreed to participate and provided written informed consent. Students undergoing pharmacological treatment for depression or anxiety were excluded. Records with incomplete anthropometric, biochemical, or questionnaire data were also excluded from the analysis.

### 2.3. Procedure and Data Collection

The procedure was carried out during the evaluation sessions of the SU SALUD-UAQ program. After receiving an explanation of the study and providing written informed consent, students underwent the routine anthropometric and biochemical assessments included in the Comprehensive Clinical Examination. Subsequently, participants completed the Garaulet Emotional Eater Questionnaire (EEQ) in a self-administered format. All information was recorded on an institutional electronic platform (susaluduaq.com) and subsequently coded into a database for statistical analysis.

### 2.4. Ethical Considerations

This study was conducted in accordance with the ethical principles established in the Declaration of Helsinki and the institutional regulations of the UAQ. The project was approved by the Bioethics Committee of the Faculty of Natural Sciences (Approval No. 008FCN2023), and the research protocol was registered at the UAQ (Registration No. 15083) on 6 January 2025. All participants received detailed information about the study objectives, procedures, and data confidentiality and provided written informed consent prior to participation. Participation was entirely voluntary and involved no additional risks, as all anthropometric and biochemical measurements were part of the routine institutional clinical evaluation. Data were handled confidentially, coded, and used exclusively for research purposes, ensuring the protection of participants’ identity and privacy.

### 2.5. Determinations

Anthropometric and body composition measurements were performed by trained professionals affiliated with the SU SALUD-UAQ program, following previously established institutional clinical protocols. Anthropometric indicators included body mass index (BMI), calculated from measured body weight and height, and waist circumference, measured at the midpoint between the lower costal margin and the iliac crest. Body composition was assessed using bioelectrical impedance analysis with an mBCA device (Model 514, SECA, Hamburg, Germany), from which body fat percentage was obtained

BMI was categorized according to World Health Organization criteria as normal weight (18.5–24.9 kg/m^2^), overweight (25.0–29.9 kg/m^2^), and obesity (≥30.0 kg/m^2^) [26]. Cut-off values for body fat percentage used to define overweight were >20% in males and >30% in females, while obesity was defined as >25% in males and >33% in females, following internationally accepted criteria [27]. Abdominal obesity was defined based on waist circumference using internationally accepted thresholds (≥80 cm for women and ≥90 cm for men), in accordance with international guidelines [28,29].

Biochemical determinations were obtained from venous blood samples collected after an 8–10 h overnight fast. The analyzed parameters included fasting glucose, triglycerides, total cholesterol, and high-density lipoprotein (HDL) cholesterol, which were measured using enzymatic methods at the laboratory of the Clinical Services Unit of the UAQ. Elevated triglycerides were defined as ≥150 mg/dL, high total cholesterol as ≥200 mg/dL, low HDL cholesterol as <50 mg/dL for women and <40 mg/dL for men, and elevated fasting glucose as ≥100 mg/dL, in accordance with internationally accepted clinical criteria [30,31].

Emotional eating was assessed using the Garaulet Emotional Eater Questionnaire (EEQ), which consists of 10 items distributed across three dimensions: disinhibition, type of food, and guilt. Each item is scored on a four-point Likert scale, resulting in a total score ranging from 0 to 30. Based on the total score, participants were classified as non-emotional eaters (0–5 points), low emotional eaters (6–10 points), emotional eaters (11–20 points), or highly emotional eaters (21–30 points). The EEQ has demonstrated adequate psychometric properties in Latin American populations, including validation studies conducted in Mexican adults, showing good internal consistency, construct validity, and factorial stability [20,21,22]. In addition, previous studies have supported its applicability in Spanish-speaking populations for the assessment of emotional eating across different weight categories [11].

To further support the use of the EEQ in the present population, a test–retest reliability analysis was conducted in a subsample of university students, demonstrating high temporal stability of the total EEQ score. This additional analysis supports the reliability of the instrument for assessing emotional eating in Mexican university students.

Information on sex, age, physical activity level (expressed as metabolic equivalents, METs), sleep duration, perceived stress, and socioeconomic status was obtained from the institutional SU SALUD-UAQ database and incorporated as covariates in the statistical analyses. Physical activity level was assessed using the long version of the International Physical Activity Questionnaire (IPAQ-Long). Total physical activity was calculated as metabolic equivalent min per week (MET-min/week) by summing walking, moderate, and vigorous activities across all domains. According to the IPAQ scoring protocol, participants were classified into three categories: low physical activity (<600 MET-min/week and not meeting criteria for higher categories), moderate physical activity (≥600 MET-min/week, including ≥3 days of vigorous activity of at least 20 min/day, or ≥5 days of moderate activity or walking of at least 30 min/day), and high physical activity (≥1500 MET-min/week of vigorous activity on ≥3 days, or ≥3000 MET-min/week from any combination of activities). These cut-off points correspond to the standard international IPAQ classification and are widely used in epidemiological studies.

Sleep duration was assessed using two self-administered questionnaires, one referring to weekdays (Monday to Friday) and another to weekends (Saturday and Sunday). For each period, average nightly sleep duration (h per night) was calculated and included as a covariate in the multivariable analyses.

Perceived stress was evaluated using the 14-item Perceived Stress Scale (PSS-14), which assesses the degree to which individuals perceive their lives as unpredictable, uncontrollable, and overloaded during the previous month. Items are rated on a five-point Likert scale (0 = never to 4 = very often). Positively worded items (items 4, 5, 6, 7, 9, 10, and 13) were reverse-scored, and a total perceived stress score was calculated by summing all items, yielding a possible range from 0 to 56, with higher scores indicating greater perceived stress. Based on the total score, participants were categorized into low (0–18 points), moderate (19–36 points), or high (37–56 points) perceived stress levels.

Socioeconomic status was obtained from the institutional SU SALUD-UAQ database and classified according to the socioeconomic level system of the Mexican Association of Market Intelligence and Opinion Agencies (AMAI), which categorizes households into hierarchical levels based on household characteristics and assets, ranging from higher (A/B) to lower (D+) socioeconomic status.

### 2.6. Statistical Analysis

Statistical analyses were performed using IBM SPSS Statistics (version 25, IBM Corp., Armonk, NY, USA). The normality of continuous variables was assessed using the Shapiro–Wilk test and visual inspection of histograms. As most variables did not meet normality assumptions, continuous variables were summarized as medians and interquartile ranges (IQR), while categorical variables were reported as absolute frequencies and percentages.

Temporal reliability (test–retest) of the EEQ was evaluated in participants with two administrations of the instrument using Spearman’s correlation coefficients (ρ) for each item (p1–p10) and for the total score. Exploratory bivariate associations between EEQ scores and anthropometric and biochemical indicators were examined using Spearman correlation analyses. Differences across emotional eating categories were assessed using the Kruskal–Wallis test with Bonferroni-adjusted post hoc comparisons, and categorical variables were compared using Pearson’s chi-square test.

To evaluate independent associations, multivariable linear regression models were fitted with anthropometric and biochemical indicators as continuous outcomes and the EEQ score as the main predictor. In addition, multivariable logistic regression models were used to estimate the odds of clinically relevant cardiometabolic outcomes according to emotional eating categories. All multivariable models were adjusted for age, sex, physical activity level (METs), sleep duration, perceived stress score, and socioeconomic status. A two-sided *p*-value < 0.05 was considered statistically significant.

### 2.7. Use of Generative Artificial Intelligence

Generative artificial intelligence (GenAI) tools were used exclusively to assist with English language editing and stylistic refinement of the manuscript. Specifically, ChatGPT (OpenAI, GPT-5) was used to improve grammar, clarity, and readability. The AI tool was not used for data analysis, data interpretation, or generation of scientific results. All outputs were critically reviewed and edited by the authors, who take full responsibility for the content of the manuscript.

## 3. Results

**Figure 1:**
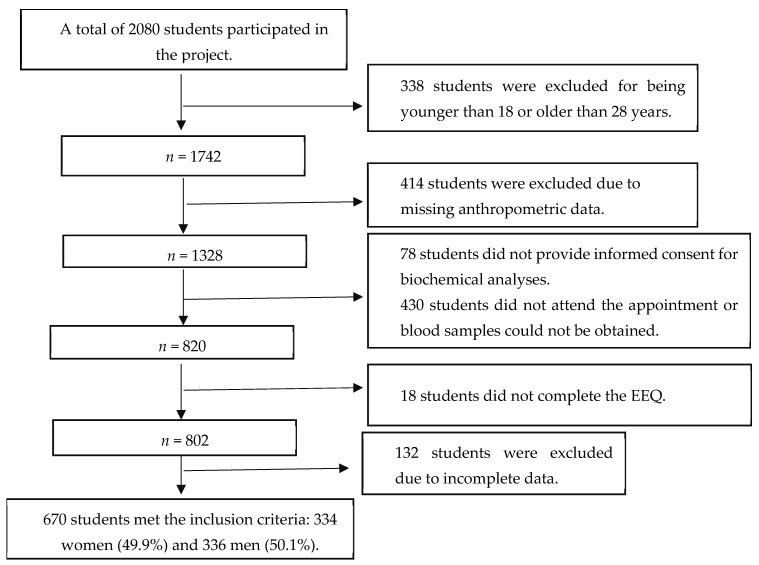
Flow diagram of participant selection.

A total of 670 university students participated in the study corresponding to a final response rate of 32.2% of the students initially considered for inclusion (2080), of whom 336 (50.1%) were men and 334 (49.9%) were women (Figure 1). The general characteristics of the study population are summarized in Table 1. The mean age was 19.32 years, with no relevant differences between sexes. Regarding nutritional status, 57.3% of participants were classified as having normal weight, 26.9% as overweight, and 8.4% as obese. The prevalence of abdominal obesity was higher in women (29.9%) than in men (24.1%). With respect to biochemical indicators, 12.4% of the total population presented elevated triglyceride levels, 13.4% had low HDL cholesterol, and 1.3% exhibited elevated glucose levels.

### 3.1. Emotional Eater Questionnaire (EEQ)

The EEQ is a validated and widely used instrument. However, due to its self-administered nature, it may be subject to recall bias and issues related to temporal stability. Therefore, to evaluate the temporal stability of the EEQ in the study population, a preliminary analysis was conducted in a subsample of students using two administrations of the instrument, with an interval ranging from 3 weeks to 2 months between the first and second assessments. From the database, participant records with more than one EEQ entry were identified and considered as test–retest pairs. This analysis included 60 university students, of whom 19 were men (30.6%) and 43 were women (69.4%). For each pair, numerical responses to the 10 questionnaire items (p1–p10) and the total score were compared. Test–retest reliability was estimated using Spearman’s correlation coefficient (ρ) between the first and second administrations, both for each individual item and for the total EEQ score (Table 2).

Overall, the EEQ items showed acceptable temporal consistency, with positive test–retest correlation coefficients ranging from low to very high magnitude (0.274–0.894), whereas the total score exhibited a very high correlation (ρ = 0.887), indicating greater stability of the global emotional eating construct compared with the variability observed at the level of individual items. These findings, obtained in Mexican university students, support the validity of the EEQ for use in this population and are consistent with the reliability reported in the original validation of the instrument [20], which described adequate test–retest stability for the total score. Table 2 summarizes the test–retest correlation coefficients for each item and for the overall questionnaire.

### 3.2. Prevalence of Emotional Eating

According to the classification of the EEQ, 33.5% of students were identified as non-emotional eaters, 31.1% as low emotional eaters, 27.6% as emotional eaters, and 7.8% as highly emotional eaters. Women showed a higher proportion in the highly emotional eater category (11.3%) compared with men (3.9%), which is consistent with previous reports describing sex differences in emotional eating behavior.

### 3.3. Anthropometric Indicators According to the Level of Emotional Eating

Anthropometric indicators by EEQ category for men and women are presented in Table 3 and Table 4, respectively. Among men, statistically significant differences were observed across all anthropometric variables (*p* < 0.001). BMI increased from 22.5 kg/m^2^ in non-emotional eaters to 28.9 kg/m^2^ in highly emotional eaters. Body fat percentage showed an increase from 18.1% to 28.7%, while waist circumference increased from 77.0 cm to 94.0 cm as the level of emotional eating classification increased. Pairwise comparisons with Bonferroni correction confirmed significant differences between groups.

Among women, statistically significant differences were also observed across all anthropometric variables (*p* < 0.001). BMI increased from 21.6 kg/m^2^ in non-emotional eaters to 26.0 kg/m^2^ in highly emotional eaters. Body fat percentage increased from 29.7% to 38.0%, while waist circumference rose from 72.0 cm to 80.0 cm. In both sexes, the observed trend showed a clear ascending pattern, whereby higher levels of emotional eating were associated with greater overall adiposity. In contrast, differences in biochemical indicators across emotional eating categories were less consistent and are described below.

### 3.4. Biochemical Indicators According to the Level of Emotional Eating

Among men, no statistically significant differences were identified for biochemical indicators, including triglycerides (*p* = 0.530), total cholesterol (*p* = 0.631), HDL cholesterol (*p* = 0.111), or glucose (*p* = 0.185), as shown in Table 3. In women (Table 4), triglyceride levels differed significantly across emotional eating categories (*p* = 0.041), showing a progressive increase from 75.0 mg/dL in non-emotional eaters to 92.0 mg/dL in highly emotional eaters. No significant differences were observed for glucose, total cholesterol, or HDL cholesterol (*p* > 0.05). These findings suggest that emotional eating may be more closely associated with metabolic alterations in women than in men.

### 3.5. Relationship Between Emotional Eating and Metabolic Risk

Overall, students classified as emotional and highly emotional eaters exhibited higher levels of general and abdominal adiposity compared with those classified as non-emotional or low emotional eaters. Differences in biochemical indicators were limited, particularly among men, whereas in women higher triglyceride levels were observed with increasing emotional eating category. These results describe distinct anthropometric and biochemical patterns across levels of emotional eating in this university population.

### 3.6. Independent Associations Between Emotional Eating and Anthropometric and Biochemical Indicators

Multivariable regression analyses were conducted to evaluate the independent associations between emotional eating and anthropometric and biochemical indicators after adjustment for potential confounding factors. As shown in Table 5, emotional eating score was significantly and positively associated with all anthropometric outcomes. Specifically, each one-point increase in EEQ score was associated with an increase of 0.221 kg/m^2^ in BMI (95% CI 0.140–0.302; *p* < 0.001), a 0.301% increase in body fat percentage (95% CI 0.152–0.450; *p* < 0.001), and a 0.565 cm increase in waist circumference (95% CI 0.370–0.759; *p* < 0.001), indicating a consistent relationship between emotional eating and both general and central adiposity.

Regarding biochemical markers, emotional eating score was independently associated with higher triglyceride concentrations (β = 1.336; 95% CI 0.118–2.555; *p* = 0.032) and lower HDL cholesterol levels (β = −0.301; 95% CI −0.525 to −0.077; *p* = 0.009), whereas no statistically significant associations were observed for total cholesterol or fasting glucose after multivariable adjustment. Overall, these findings indicate that emotional eating is independently associated with increased adiposity and selected lipid alterations, even after accounting for sociodemographic and behavioral covariates.

In sex-stratified multivariable analyses, emotional eating score remained independently associated with anthropometric indicators in both men and women after adjustment for age, physical activity level, sleep duration, stress, and socioeconomic status. Higher EEQ scores were consistently associated with increased BMI, body fat percentage, and waist circumference in both sexes, confirming a robust relationship between emotional eating and both general and central adiposity. With respect to biochemical indicators, emotional eating score was independently associated with higher triglyceride concentrations and lower HDL cholesterol levels in women, whereas no statistically significant associations were observed for total cholesterol or fasting glucose after multivariable adjustment. In men, no independent associations were identified between emotional eating score and biochemical markers. These findings suggest sex-specific differences in the metabolic correlates of emotional eating, with stronger associations observed among women.

Multivariable logistic regression analyses were performed to estimate the odds of clinically relevant cardiometabolic outcomes according to emotional eating categories. As shown in Table 6, students classified as emotional or highly emotional eaters exhibited significantly higher odds of excess adiposity compared with non- and low-emotional eaters. Emotional eating was independently associated with nearly threefold higher odds of overweight or obesity (OR = 2.97; 95% CI 1.79–4.92; *p* < 0.001) and 2.7-fold higher odds of elevated body fat percentage (OR = 2.70; 95% CI 1.65–4.44; *p* < 0.001).

Notably, the strongest association was observed for abdominal obesity, with emotional eaters presenting more than fourfold increased odds (OR = 4.27; 95% CI 2.48–7.36; *p* < 0.001), indicating a particularly robust link between emotional eating and central fat accumulation. In addition, emotional eating was independently associated with low HDL cholesterol levels (OR = 2.50; 95% CI 1.31–4.77; *p* = 0.006), whereas no statistically significant association was identified for elevated triglycerides in the dichotomous model.

Overall, the results presented in Table 6 indicate that emotional eating substantially increases the likelihood of clinically relevant obesity phenotypes and selected lipid abnormalities, independent of behavioral and sociodemographic covariates, with stronger associations observed among women.

## 4. Discussion

Emotional eating has been increasingly recognized as a relevant behavioral factor associated with excess adiposity during early adulthood. In this context, the present study shows that more than one third of the university population exhibited moderate to high levels of emotional eating, with a higher proportion of women classified as highly emotional eaters. Importantly, multivariable analyses revealed a consistent gradient between the severity of emotional eating and indicators of general and central adiposity in both sexes, independent of age, physical activity, sleep duration, perceived stress, and socioeconomic status. In contrast, independent associations with biochemical lipid parameters were limited and observed exclusively among women. Taken together, these findings are consistent with recent literature and support the relevance of considering the emotional dimension of eating as a behavioral factor associated primarily with adiposity-related outcomes, with more modest and sex-specific associations observed for selected metabolic markers in young adults [7,14,23].

Eating behavior is shaped by the interaction of internal factors, such as emotions, stress, impulsivity, and affective disorders, and external influences, including the university food environment, food advertising, irregular schedules, and sedentary lifestyles. Within this context, sustained exposure to ultra-processed foods, sleep deprivation, and academic stress may increase the likelihood of using food as a coping strategy, thereby promoting dysregulated eating patterns such as emotional eating and binge eating [1,2,32]. From a behavioral and physiological perspective, emotional eating reflects a dysregulation in the balance between homeostatic mechanisms involved in energy regulation and hedonic systems that drive reward-seeking behavior. Under conditions of stress or emotional distress, hedonic processes may predominate, favoring food intake beyond physiological energy requirements and contributing to the accumulation of general and central adiposity. This interpretative framework is consistent with evidence from university populations, in which emotional eating has been associated with higher levels of overall and abdominal adiposity [1,33].

The prevalence observed in the present study is comparable to that reported in international investigations, which indicate that approximately 30% to 40% of university students exhibit moderate to high levels of emotional eating [6]. In Mexico, previous studies have documented that dysfunctional eating behaviors, including eating in response to emotional states, are associated with overweight, obesity, and anxiety–depressive symptoms among university students [13,14,34]. The higher prevalence of emotional eating observed among women in this study is consistent with findings in university and adult populations and suggests a greater female vulnerability to using food as a strategy for affect regulation [1,2,6]. This sex difference may reflect the interaction of psychological, sociocultural, and biological factors. Women tend to report higher emotional awareness and internalization of stress, which may increase reliance on food for emotion regulation, while sociocultural pressures related to body image and weight control may further reinforce dysregulated eating patterns. In addition, sex-related differences in stress reactivity and neuroendocrine responses—particularly involving cortisol and reward-related dopaminergic pathways—have been proposed as contributing mechanisms. Together, these factors may help explain why emotional eating appears more prevalent and more strongly associated with adiposity-related outcomes among women [1,2,6].

Foods rich in simple sugars, fats, and salt strongly activate dopaminergic reward circuits, which may transiently reduce emotional distress and reinforce repeated consumption. These foods can also elicit rapid endocrine responses that modulate cortisol, serotonin, and dopamine signaling, thereby favoring a positive energy balance. This behavioral and physiological context may help explain the observed associations between emotional eating and increased adiposity, as well as the more limited and sex-specific associations with lipid alterations observed in this study, particularly among women [1,6].

The positive relationship between emotional eating and adiposity observed in the present study is consistent with findings reported in recent reviews. Chew et al. [35] indicate that emotional eating is consistently associated with higher BMI and energy-dense dietary patterns, which favor excess energy intake. Other studies have shown that behaviors such as emotional or disinhibited eating predict an increased risk of weight gain, particularly among younger individuals [36]. Together, these findings suggest that the association between emotional eating and excess adiposity may already be established during early adulthood, including the university stage. From an integrative perspective, emotion-driven overeating may contribute to a self-perpetuating cycle in which increased food intake promotes weight gain and body dissatisfaction, which in turn may exacerbate emotional distress and reinforce the use of food as a coping strategy [2,5]. This framework supports the interpretation of emotional eating as a relevant behavioral factor linked primarily to adiposity-related outcomes in young adults.

The adjusted analyses further support this interpretation by demonstrating that emotional eating is independently associated with greater adiposity in both sexes, which is consistent with previous studies reporting similar associations in adolescents and young adults [37,38]. In women, emotional eating was also independently associated with less favorable lipid profiles, suggesting that this eating pattern may be linked to early and sex-specific metabolic alterations. Recent evidence indicates that the coexistence of increased adiposity, poor diet quality, and emotional eating is associated with a higher cardiometabolic vulnerability among young women [37,38]. In contrast, no independent associations with biochemical indicators were observed in men, which may be partly explained by sex-related differences in metabolic responses to stress and emotionally driven food intake, as well as differences in hormonal and neuroendocrine regulation [39]. In line with these findings, multivariable logistic regression analyses indicated that students classified as emotional or highly emotional eaters had significantly higher odds of presenting general and central adiposity. Notably, the strongest associations were observed for abdominal obesity, particularly among women, even after adjustment for age, physical activity level, sleep duration, perceived stress, and socioeconomic status.

From a mechanistic perspective, emotional eating may reflect an imbalance between homeostatic and hedonic regulation of food intake, whereby reward-driven processes can override physiological hunger signals under conditions of emotional distress [1,2,5]. This framework provides a plausible explanation for the consistent associations observed between emotional eating and increased general and central adiposity in young adult populations.

From a psychological perspective, the present findings are consistent with accumulating evidence linking emotional eating to higher levels of perceived stress, anxiety, depressive symptoms, and poorer sleep quality [32,36]. Previous studies have shown that university students experiencing greater emotional distress are more likely to rely on ultra-processed foods and exhibit less healthy dietary patterns [13,16]. Within this framework, emotional eating may represent one component of a broader set of maladaptive coping behaviors adopted in response to emotional and academic demands during the university stage.

A major strength of this study is the use of the EEQ developed by Garaulet et al. [20], a validated instrument specifically designed to assess emotional eating across populations with a wide range of body weight variability. In addition, the inclusion of anthropometric and biochemical indicators obtained through standardized clinical procedures enhances the robustness and reliability of the findings.

Nevertheless, several limitations should be acknowledged. First, the cross-sectional design precludes causal inference; therefore, it is not possible to determine whether emotional eating contributes to increased adiposity or whether it emerges as a response to weight-related concerns or academic stress. Second, the sample was drawn from a single university, which may limit the generalizability of the findings to other university populations or sociocultural contexts. In addition, emotional eating was assessed using a self-reported questionnaire, which may be subject to recall and social desirability bias. Although the EEQ is a validated instrument and showed high temporal stability in the present study, self-reported responses may not fully capture habitual eating behaviors or emotional states over time. Finally, dietary intake was not assessed, which may have influenced the observed associations. Although physical activity level, sleep duration, perceived stress, and socioeconomic status were included as covariates in the multivariable analyses, residual confounding related to unmeasured aspects of diet quality and eating context cannot be excluded. In addition, although the final analytical sample represented 32.2% of the students initially enrolled in the institutional health evaluation during the academic period, it should be noted that exclusions were primarily related to predefined eligibility criteria and incomplete clinical or questionnaire data rather than systematic refusal to participate. The resulting sample comprised 670 students from multiple campuses and academic programs, with balanced sex distribution, which strengthens internal validity. Nevertheless, the possibility of selection bias cannot be entirely excluded, particularly if students who completed all components of the clinical evaluation differ systematically from those who did not.

This study has relevant implications for university public health. The identification of a high prevalence of emotional eating, together with its consistent association with adiposity and more limited lipid alterations observed among women, suggests that emotional factors may represent an important component to consider within university health promotion initiatives. Preventive approaches that integrate nutrition education, emotional regulation, and stress management may be particularly relevant in this context. Mindfulness-based eating interventions, for example, have shown potential for reducing impulsive eating behaviors and improving individuals’ relationship with food [16]. In addition, these findings may inform university-level strategies aimed at promoting healthier food environments, including efforts to reduce exposure to ultra-processed foods [40], particularly in countries with a high prevalence of overweight and obesity. Overall, this study provides context-specific evidence in a young Mexican population, indicating that emotional eating is a frequent behavior associated primarily with greater adiposity in both sexes and with less favorable lipid profiles in women. These findings underscore the importance of considering the emotional dimension of eating within future obesity prevention and health promotion strategies in university settings.

## 5. Conclusions

The present study shows that emotional eating is a highly prevalent behavior among the evaluated university students, with more than one third of the population classified as emotional or highly emotional eaters and a higher prevalence observed among women. The findings indicate a consistent association between emotional eating and higher levels of adiposity—including BMI, body fat percentage, and waist circumference—in both sexes. In addition, associations between emotional eating scores and selected lipid parameters were observed among women, suggesting the presence of early and sex-specific metabolic correlates linked to this eating pattern.

Overall, these findings indicate that emotional eating represents a relevant behavioral factor associated primarily with excess adiposity in young university populations, with more limited associations observed for metabolic markers. The results support the relevance of incorporating the assessment of emotional eating into university health promotion strategies focused on nutrition education, stress management, and emotional regulation. In addition, they may inform institutional efforts aimed at fostering healthier food environments and reducing exposure to ultra-processed foods. Finally, longitudinal studies are warranted to clarify the directionality of the observed associations and to evaluate the effectiveness of interventions designed to address emotional eating behaviors over time.

## Figures and Tables

**Table 1:** General characteristics of the study population.

Variable	Male	Female	Total
Population	336	334	670
Age (years)	19.48 (SD = 4.3)	19.16 (SD = 1.7)	19.32
BMI (kg/m^2^)	24.15 (SD = 4.4)	23.79 (SD = 4.2)	23.97 (SD = 4.3)
BMI categories	Underweight: 6.3% Normal: 58% Overweight: 27.4% Obesity: 8.3%	Underweight: 8.7% Normal: 56.6% Overweight: 26.3% Obesity: 8.4%	Underweight: 7.5% Normal: 57.3% Overweight: 26.9% Obesity: 8.4%
Body fat %	21.29 (SD = 8.6)	32.06 (SD = 7.2)	26.66 (SD = 9.6)
Body fat % (categories)	High: 17.6% Obesity: 32.8%	High: 22.2% Obesity: 26%	High: 19.9% Obesity: 30.1%
Waist circumference (cm)	82.22 (SD = 11.8)	75.65 (SD = 9.4)	78.95 (SD = 11.1)
	Abdominal Obesity: 24.1%	Abdominal Obesity: 29.9%	Abdominal Obesity: 27%
High triglycerides	14%	10.8%	12.4%
Total cholesterol (mg/dL)	150.62 (SD = 29.2)	150.48 (SD = 27.7)	150.55 (SD = 28.5)
High total cholesterol (%)	5.4%	3.9%	4.6%
HDL cholesterol (mg/dL)	44.0 (SD = 10.6)	48.9 (SD = 11.6)	46.5 (SD = 11.4)
Low HDL cholesterol (%)	18.5%	8.4%	13.4%
Glucose (mg/dL)	76.11 (SD = 14.9)	71.46 (SD = 8.2)	73.79 (SD = 12.3)
High fasting glucose (%)	3%	0.6%	1.3%
Physical activity (MET-min/week)	2169.11 (SD = 1696.9)	1562.03 (SD = 1529.5)	1853.06 (SD = 1638.8)
High physical activity (%)	44.3%	31.7%	38.1%
Moderate physical activity (%)	16.7%	26.9%	21.8%
Low physical activity (%)	15.2%	24.6%	19.9%
Average weekday sleep duration (h/night) *	4.1 (SD = 3.4)	4.0 (SD = 3.4)	4.0 (3.4)
Average weekend sleep duration (h/night) *	8.7 (SD = 1.3)	9.2 (SD = 1.5)	8.9 (SD = 1.4)
Hours of adequate sleep (%)	24.7	23.4%	24.0%
Socioeconomic level (%) **			
A/B	56.5%	48.8%	52.7%
C	9.8%	13.25%	11.5%
C−	3.6%	6.3%	4.9%
C+	22.3%	22.5%	22.4%
D+	3.3%	4.5%	3.9%
Self-perceived stress level (%) ***			
High	5.1%	16.8%	10.9%
Low	25.9%	10.8%	18.4%
Moderate	64.3%	67.7%	66%

* Sleep duration was assessed using two separate self-administered questionnaires covering weekdays (Monday to Friday) and weekends (Saturday and Sunday). Values represent the average number of hours slept per night during each period. ** Perceived stress was assessed using the 14-item Perceived Stress Scale (PSS-14; Escala de Estrés Percibido, EEP). Total scores range from 0 to 56, with higher scores indicating greater perceived stress, and were categorized as low (0–18 points), moderate (19–36 points), or high (37–56 points). *** Socioeconomic level was classified according to the institutional criteria used in the SU SALUD-UAQ program, based on the Mexican Association of Market Intelligence and Opinion Agencies (AMAI) socioeconomic level classification, which categorizes households into hierarchical levels ranging from higher (A/B) to lower (D+) socioeconomic status.

**Table 2:** Test–retest reliability of the EEQ in university students (*n* = 62).

Item	Description (Abbreviated)	Spearman Correlation Coefficient (ρ)	*p*-Value
P1	The scale affects your mood	0.558 **	0.000
P2	Cravings for specific foods	0.274 *	0.031
P3	Difficulty stopping eating sweets	0.771 **	0.000
P4	Controlling the amount of certain foods	0.721 **	0.000
P5	Eating when stressed, angry, or bored	0.894 **	0.000
P6	Eating more and more uncontrollably when alone	0.659 **	0.000
P7	Feeling guilty when eating “forbidden” foods	0.753 **	0.000
P8	Greater lack of control at night when tired from school	0.539 **	0.000
P9	Loss of control when breaking your diet	0.521 **	0.000
P10	Feeling that food controls you	0.618 **	0.000
Total Emotional Eater Questionnaire score (10 items)		0.887 **	0.000

Spearman correlation coefficients (ρ) were calculated between the first and second administrations of the Emotional Eater Questionnaire (EEQ) to assess test–retest reliability. * *p* < 0.05; ** *p* < 0.01.

**Table 3:** Anthropometric and biochemical indicators by emotional eating category in men (*n* = 336).

Variable	Non-Emotional (*n* = 119)	Low Emotional (*n* = 112)	Emotional (*n* = 83)	Very Emotional (*n* = 13)	*p*-Value *
BMI (kg/m^2^)	22.5 (4.8) ^a^	23.8 (5.2) ^ab^	25.4 (5.6) ^b^	28.9 (7.4) ^c^	0.000
Body fat (%)	18.1 (10.9) ^a^	21.5 (11.9) ^ab^	23.6 (11.3) ^bc^	28.71 (17.9) ^c^	0.000
Waist circumference (cm)	77.0 (14.0) ^a^	82.0 (14.8) ^ab^	87.0 (16.0) ^bc^	94.0 (25.0) ^c^	0.000
Triglycerides (mg/dL)	87.0 (49.0)	84.5 (51.0)	87.0 (70.0)	97.0 (86.0)	0.530
Total cholesterol (mg/dL)	151.0 (36.0)	151.0 (41.0)	145.0 (45.0)	147.0 (57.0)	0.631
HDL cholesterol (mg/dL)	45.0 (14.0)	44 (13.0)	42.0 (15.0)	38 (11.0)	0.111
Glucose (mg/dL)	76.0 (13.0)	74.0 (14.0)	73.0 (13.0)	69.0 (14.0)	0.185

Emotional eating categories were defined according to the Garaulet Emotional Eater Questionnaire. Data are presented as median and interquartile range (IQR). * Kruskal–Wallis test. Different superscript letters (a, b, c) indicate statistically significant differences (*p* < 0.05) between emotional eating categories within the same variable, based on Bonferroni-adjusted post hoc multiple comparisons. Categories sharing at least one letter do not differ significantly from each other, whereas categories with different letters indicate statistically significant differences.

**Table 4:** Anthropometric and biochemical indicators by emotional eating category in women (*n* = 334).

Variable	Non-Emotional (*n* = 101)	Low Emotional (*n* = 92)	Emotional (*n* = 98)	Very Emotional (*n* = 38)	* *p*-Value
BMI (Kg/m^2^)	21.6 (5.1) ^a^	22.2 (5.5) ^a^	24.8 (6.1) ^b^	26.0 (6.1) ^b^	0.000
Body fat (%)	29.7 (9.5) ^a^	30.8 (9.4) ^ab^	34.8 (8.9) ^b^	38.0 (8.4) ^c^	0.000
Waist circumference (cm)	72.0 (11.0) ^a^	73.0 (12.0) ^a^	78.0 (13.3) ^b^	80.0 (14.5) ^c^	0.000
Triglycerides (mg/dL)	75.0 (40.0) ^a^	77.0 (43.0) ^a^	89.5 (53.0) ^a^	92.0 (43.0) ^ab^	0.041
Total cholesterol (mg/dL)	149.0 (41)	149.0 (30.0)	152.5 (32.0)	153.0 (42.0)	0.153
HDL cholesterol (mg/dL)	49.0 (18.0)	49.0 (14)	47.0 (16.0)	43.0 (19.0)	0.186
Glucose (mg/dL)	72.0 (12.0)	71.0 (9.0)	72.0 (12)	70.5 (13)	0.910

Emotional eating categories are based on the Garaulet Emotional Eater Questionnaire. Data are presented as median and interquartile range (IQR). * Kruskal–Wallis test Different superscript letters (a, b, c) indicate statistically significant differences (*p* < 0.05) between emotional eating categories within the same variable, based on Bonferroni-adjusted post hoc multiple comparisons. Categories sharing at least one letter do not differ significantly from each other, whereas categories with different letters indicate statistically significant differences.

**Table 5:** Associations between emotional eating score and anthropometric and metabolic outcomes.

Outcome	β (95% CI)	*p*-Value	Adjusted R^2^
BMI (kg/m^2^)	0.221 (0.140–0.302)	<0.001	0.115
Body fat (%)	0.301 (0.152–0.450)	<0.001	0.369
Waist circumference (cm)	0.565 (0.370–0.759)	<0.001	0.213
Triglycerides (mg/dL)	1.336 (0.118–2.555)	0.032	0.010
HDL cholesterol (mg/dL)	−0.301 (−0.525 to −0.077)	0.009	0.080
Total cholesterol (mg/dL)	0.549 (−0.041–1.138)	0.068	0.009
Fasting glucose (mg/dL)	−0.056 (−0.330–0.218)	0.690	0.049

Multivariable multiple linear regression. Models adjusted for sex, physical activity level, weekday sleep duration, weekend sleep duration, stress score, and socioeconomic status. β represents unstandardized regression coefficients. CI: Confidence interval.

**Table 6:** Multivariable logistic regression analysis of the association between emotional eating and cardiometabolic risk outcomes in university students.

Outcome	OR (95% CI)	*p*-Value
Overweight/Obesity (BMI)	2.97 (1.79–4.92)	<0.001
Elevated Body Fat (%)	2.70 (1.65–4.44)	<0.001
Abdominal Obesity (Waist Circumference)	4.27 (2.48–7.36)	<0.001
Low HDL Cholesterol	2.50 (1.31–4.77)	0.006
Elevated Triglycerides	1.50 (0.78–2.88)	0.224

Multivariable binary logistic regression model adjusted for age, sex, physical activity level, sleep duration, stress, and socioeconomic status. OR: odds ratio; CI: confidence interval.

## Data Availability

The original contributions presented in this study are included in the article. Further inquiries can be directed to the corresponding author.
